# Facile Synthesis of Cobalt Oxide as an Efficient Electrocatalyst for Hydrogen Evolution Reaction

**DOI:** 10.3389/fchem.2020.00386

**Published:** 2020-05-07

**Authors:** Yinbo Wu, Ruirui Sun, Jian Cen

**Affiliations:** ^1^Guangdong Polytechnic Normal University, Guangzhou, China; ^2^Safety and Environmental Protection Division of Jilin Petrochemical Company, PetroChina, Jilin, China; ^3^The Key Laboratory for Smart Building Equipment Integration of Guangzhou, Guangzhou, China

**Keywords:** hydrogen evolution reaction, electrocatalysts, cobalt oxide, facile synthesis, hydrothermal

## Abstract

Hydrogen evolution reaction (HER) is receiving a lot of attention because it produces clean energy hydrogen. Catalyst is the key to the promotion and application of HER. However, the precious metal catalysts with good catalytic performance are expensive, and the preparation process of non-precious metal catalysts is extremely complicated. The simple preparation process is the most important problem to be solved in HER catalyst development. We synthetized cobalt oxide (CoO_x_) catalyst for HER through a simple hydrothermal process. The CoO_x_ catalyst shows excellent HER catalytic activity. Characterization results reveal that there are a great deal of surface hydroxyl groups or oxygen vacancy on the surface of CoO_x_ catalyst. In alkaline media the CoO_x_ catalyst shows an over-potential of 112 mV at 20 mA cm^−2^ and a small Tafel slope of 94 mV dec^−1^. This paper provides a simple and easy method for HER catalyst preparation.

## Introduction

At present, the world's energy consumption mainly comes from the expending of fossil energy such as coal, oil, and natural gas. The burning of fossil energy brings two problems (Ojha et al., 2018). First, fossil energy is a non-renewable resource, and it will be exhausted in the near future. Second, the burning of fossil energy generates fearful environmental questions such as haze and chemical rain (Chandrasekaran et al., 2019). H_2_ is a hopeful clean and renewable source of energy to resolve the obstacle of fossil fuel exhaustion and environment increasingly being destroyed (Chen et al., 2013; Zhao et al., 2020). In recent years, hydrogen production from electrolyzed water has attracted scientists from all over the world as an emerging method of hydrogen production (Morales-Guio et al., 2014). Scientists have discovered that Pt-based precious metal materials are the best electrocatalysts for HER. However, platinum-based noble metal materials limit their wide application in electrocatalysts due to scarce resources and high prices (Zou and Zhang, 2015; Liu et al., 2020). Consequently, it is necessary to exploit no-noble metal catalysts which are inexpensive and stable in activity. Because of their Pt-like catalytic behaviors for HER, well electrocatalysts originated from the most abundant elements (Co, Fe, Mo, Ni, Ti, W, and so on) has experienced rapid development over the past decade (Jung et al., 2014; Kuznetsov et al., 2019; Liu et al., 2020). Scientists design and develop various catalysts including transition metal oxides (CoO, Fe_3_O_4_, MoO_2_, TiO_2_, WO_2_) (Park and Kolpak, 2019; Protsenko et al., 2019; Qian et al., 2019; Li L. et al., 2020; Li S. et al., 2020), metal sulfides (CoS, CuS, FeS_2_, MoS_2_, NiS_2_, V_3_S_4_, WS_2_) (Li et al., 2018; Shi et al., 2019; Singh et al., 2019; Wang Y. et al., 2019; Cao et al., 2020; Hao et al., 2020; Thangasamy et al., 2020), metal carbides (MoC_2_, WC) (Ji et al., 2018; Hussain et al., 2019), metal phosphides (CoP, NiP, FeP, WP, MoP) (Ojha et al., 2017; Zhang C. et al., 2018; Wang P. et al., 2019; Ji et al., 2020; Lin et al., 2020), metal selenides (CoSe_2_, MoSe_2_, NiSe_2_, WSe_2_) (Wang et al., 2012, 2020; Chen et al., 2020; Nam et al., 2020) and metal boron (Huang et al., 2019; Wang A. et al., 2019).

In order to obtain these prominent catalysts, scientists have explored multifarious methods. In addition to using ultrasonic vibration, hydrothermal, calcination and other methods, it also needs to undergo steps such as sulfurization, selenization, phosphating or other methods. As we all know, sulfur, selenium, phosphorus and so on are all toxic and flammable under certain conditions, which hinders the widespread use of these catalysts for HER. In order to enable the hydrogen evolution reaction to be promotion and application, its catalyst preparation must be simple and easy to achieve industrial production. Therefore, a simple and easy preparation method is one of the important tasks in the development of HER catalysts.

Among the many metal oxide catalysts, the cobalt oxide possesses a favorable activity and stability for HER (Wang et al., 2016). Because of its unique electronic state, the cobalt oxides demonstrate the good electrocatalytic activity (Zhang X. et al., 2018). Inspired by the above-mentioned, we herein have synthesized octahedral cobalt oxide particles using Co foam through the simple hydrothermal method without adding any other substances. Impressively, the resultant catalyst reveals favorable electrocatalytic performances and excellent long-term stability for the HER.

## Experimental Details

### Synthetic Process

All chemical substances in the article were of analytical grade and utilized as obtained without purification treatment. Co foam was disposed by ultrasonic vibration in 1.5 M hydrochloric acid solution, acetone and secondary distilled water for 30 min, respectively. Then the treated Co foam and some redistilled water were put into a Teflon-lined autoclave (rated capacity 100 mL). The autoclave was healed and placed at 200°C for 24 h. After the hydrothermal reaction accomplished, the autoclave was naturally cooled naturally to indoor temperature. After moisturized with filter paper, the Co foam was subjected to heat in a vacuum tube furnace at 250°C for 30 min. The sample was obtained after cooling and named Co1.

### Characterization

XRD data was obtained by a Bruker D8 Advance X-ray diffractometer (Cu Ka, l ¼ 1.5418 Å). The surface morphology and microstructure were determined by scanning electron microscope (FE-SEM, JEOL JSM-6700F) and transmission electron microscope (TEM, Tecnai G2 F20). X-ray photoelectronspectroscopy (XPS) measurements were examined by a Phi V5000 X-ray photoelectron spectrometer with Al–Kα radiation (hν = 1486.6 eV). Inductively coupled plasma-atomic emission spectrometry (ICP-AES) was conducted on a Leeman PROFILE SPEC.

### Electrochemical Measurements

All the electrochemical measurements were conducted on a CHI 760E electrochemical workstation (Shanghai Chenhua) in a standard three-electrode system. The Co foam sample was used as the working electrode, a graphite plate was used as the counter electrode and the saturated calomel electrode (SCE) was used as the reference electrode. For all electrochemical tests, the electrolyte (1.0 M KOH) was continuously bubbled by high-purity nitrogen during the experiment. All linear sweep voltammetry (LSV) tests were conducted with a uniform scan rate of 5 mV s^−1^. Electrochemical impedance spectroscopy (EIS) was performed in the frequency scope from 10^5^ to 0.01 Hz under open circuit potential. Cyclic voltammogram (CV) was detected at different scan rates (20, 40, 60, 80, 100, 120 mV s^−1^). The stability measurements were characterized by chronopotentiometric method. Furthermore, all potentials mentioned in the article were calibrated vs. reversible hydrogen electrode (RHE) according to the formula *E*(*RHE*) = *E*(*SCE*)+0.0591pH+0.2415−0.000761^*^(*T*−298.15).

## Results and Discussion

**Figure 1:**
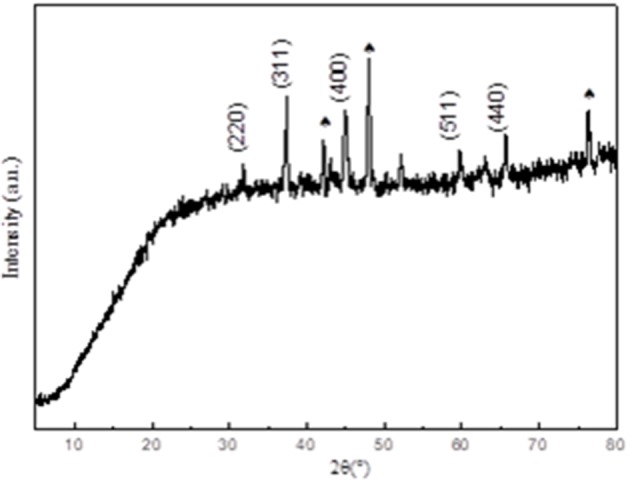
XRD patterns of Co1.

Figure 1 shows the X-ray diffraction (XRD) characterization of the Co1catalyst. The main components of the material can be derived from the XRD test results. As shown in Figure 1, the diffraction angles of 31°, 37°, 45°, 59°, and 65° in the XRD curve corresponds to the standard diffraction peak of Co_3_O_4_ (PDF.43-1003), respectively. The spades of diffraction peaks correspond to the X-ray diffraction peak of untreated cobalt foam (Figure S1). The diffraction peak of 52° is also attributed to untreated cobalt foam. And the peak that appears at 62° belong to CoO.

**Figure 2:**
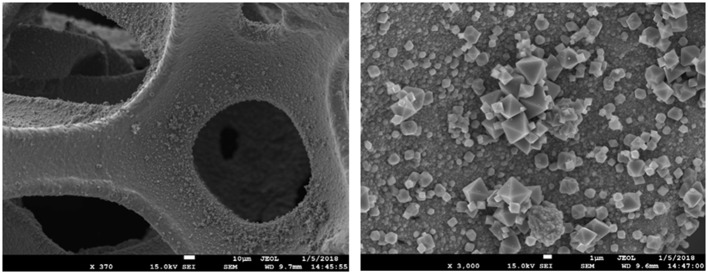
SEM images of Co1.

**Figure 3:**
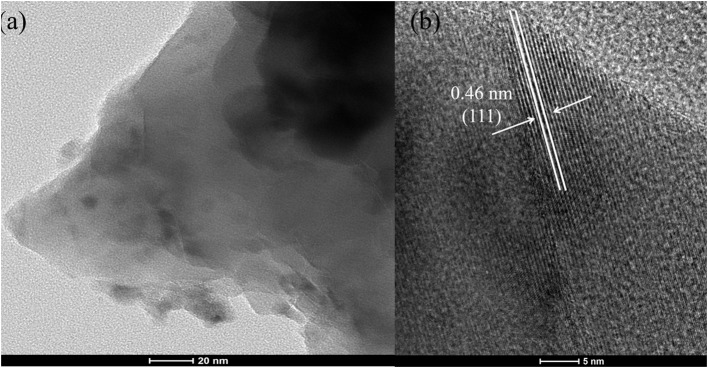
**(a)** TEM image of Co1 catalyst; **(b)** High-resolution TEM image of Co1 catalyst.

Figure 2 shows the SEM image of Co1. As shown in the picture, octahedral CoO_x_ particles covered on the skeleton of cobalt foam. These particle sizes are mostly about 1 to 3 μm. The high-resolution TEM pictures and of the particles peeled from Co1 is shown in Figure 3. From the lattice fringes reveal an interplanar distance of 0.46 nm, which belong to the (111) plane of Co_3_O_4_. The EDS data (Figure S2 and Table S2) indicates that the Co element content should be 64 wt%. The result obtained by EDS is nearly equal to that of ICP-AES (Co 65 wt%). This is probably due to a massive hydroxyl groups on sample surface.

**Figure 4:**
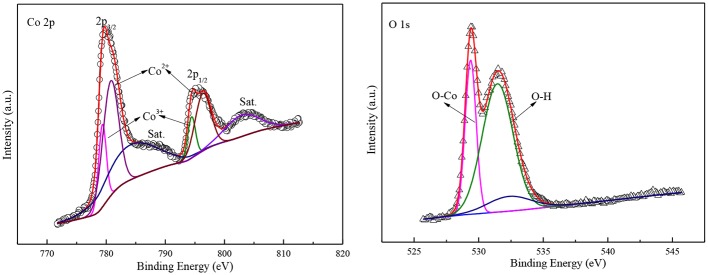
XPS spectra of Co 2p and O 1s.

More information on the composition and valence state of the sample was obtained by XPS. The 2p spectra of Co and 1s spectra of O are shown in Figure 4 which calibrated with a C 1s peak of 284 eV. The high-resolution core spectrum for Co 2p, where the peaks at 780 and 798 eV are belonged to Co 2p3/2 and 2p1/2 peak in Co_3_O_4_ (Cheng et al., 2013). The two Co 2p peaks can be deconvoluted into four sub-peaks at 778, 781, 795, and 799 eV, representing signatures of ${\text{Co}}_{3}^{+}$ and ${\text{Co}}_{2}^{+}$, respectively (Xu et al., 2016). These values are in accordance with the Co2p state, suggesting the Co 2p is derived from CoO. In addition, there are two satellite peaks at 785 eV and 805 eV in Figure 4 indicate that the most of Co element in the end product is Co^2+^ cation (Lv et al., 2018). This represents there is a high concentration of ${\text{Co}}_{2}^{+}$ on the surface of the Co_3_O_4_. Therefore, it can come to the conclusion that there is numerous a lot of oxygen vacancy to neutralize electrons for HER.

For the high-resolution O 1s spectrum, the peak centered at 528 eV correspond to the lattice oxygen in Co_3_O_4_, and the peak at 532 eV is related to hydroxyl groups or oxygen vacancy on surface. Moreover, the bread peak at 533.5 eV could be ascribed to absorbed oxygen on the surface of Co1 catalyst. The peak intensity of hydroxyl groups is much greater than the peak intensity of absorbed oxygen, which suggest that there are a great deal of surface hydroxyl groups and oxygen vacancy on the sample surface. According to the reported paper, the hydroxyl groups on catalyst surface could promote the decomposition of H_2_O by attenuating O-H bond (Xu et al., 2016). Moreover, the oxygen vacancy can boost the conductivity of HER catalyst and, boost the adsorption of H_2_O molecules. Thus, the cobalt oxide particles possess good catalytic performance for HER.

**Figure 5:**
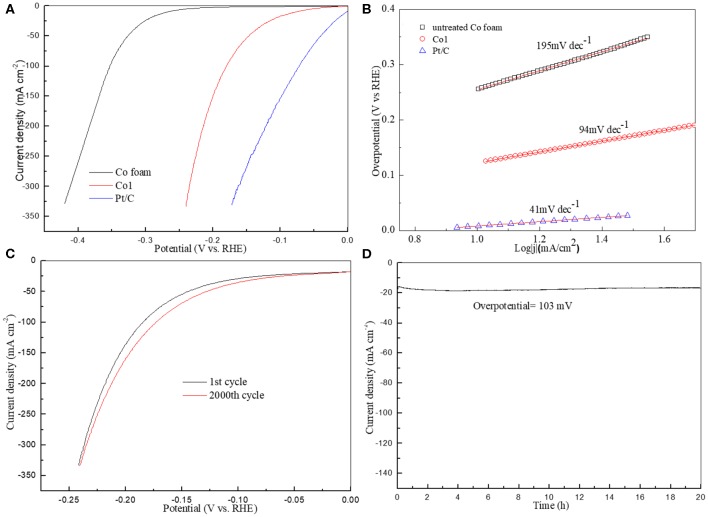
**(A)** Polarization curves of untreated Co foam, Co1 and Pt/C; **(B)** Corresponding Tafel plots; **(C)** The first and 2000th cycle polarization curves of Co1; **(D)** Stability test for Co1.

The HER catalytic activities of the Co1 catalyst were investigated in 1.0 M KOH with a representative three-electrode system. Comparison of polarization curves is provided in Figure 5A together with untreated Co foam and commercial Pt/C. All of the polarization curves are ohmic potential drop (iR)-corrected. The Co1 catalyst demonstrates a dramatically high activity with an onset potential of ~40 mV 10 mA cm^−2^ (Figure 5A), which was comparable to the reported cobalt oxides measured in 1.0 M KOH, such as Co_3_O_4_ nanosheets (49 mV), CoO_x_ (85 mV) (Table S1). When the HER current density achieves to 20 mA cm^−2^, but only a small overpotantial as 112 mV. The cobalt oxide was shown to promote the dissociation of H_2_O on account of the stronger electrostatic affinity of OH^−^ to Co^2+^ and Co^3+^ in alkaline medium.

In general, according to the rate-determining step, the HER mechanism can be fall into two basic types, Volmer–Tafel and Volmer–Heyrovsky mechanism. In the Volmer–Tafel theory, firstly the initial M–H bond is formed in Volmer step, secondly followed the dimerization of two adsorbed H in Tafel step. But in the Volmer–Heyrovsky mechanism, the adsorbed H reacts with the proton source in alkaline solution in Heyrovsky step (Liu et al., 2017). Tafel plot is often useful to reflect the electrode catalytic performance and evaluate the rate determining step of the HER mechanism. The Tafel plot for each of the polarization curves in Figure 5A is presented in Figure 5B. The Tafel slope of Co1 is 94 mV dec^−1^, which is superior to that of untreated Co foam (195 mV dec^−1^). Low Tafel slope indicates the catalyst possess strong catalytic activity. It indicates that the HER might be proceeded via the Volmer–Heyrovsky mechanism. Stability is significant indicator to evaluate the performance of HER catalyst. As shown in Figures 5C,D, the stability measurements of the Co1 sample are inducted by linear sweep voltammetry and chronoamperometry. The minimal attenuation of the polarization curves in Figure 5C after 2000 cycles signifies that Co1 catalyst possesses favorable electrochemical stability in alkaline medium. As shown in SEM images of Co1 after the stability measurements (Figure S4), many gaps appeared on the smooth surface of octahedral catalyst after the 2000 cycles linear sweep voltammetry test. Additionally, as shown in Figure 5D, Co1 catalyst can keep a stead current density at 16.5 mA cm^−2^ at a given over-potential (103 mV) for 20 h.

**Figure 6:**
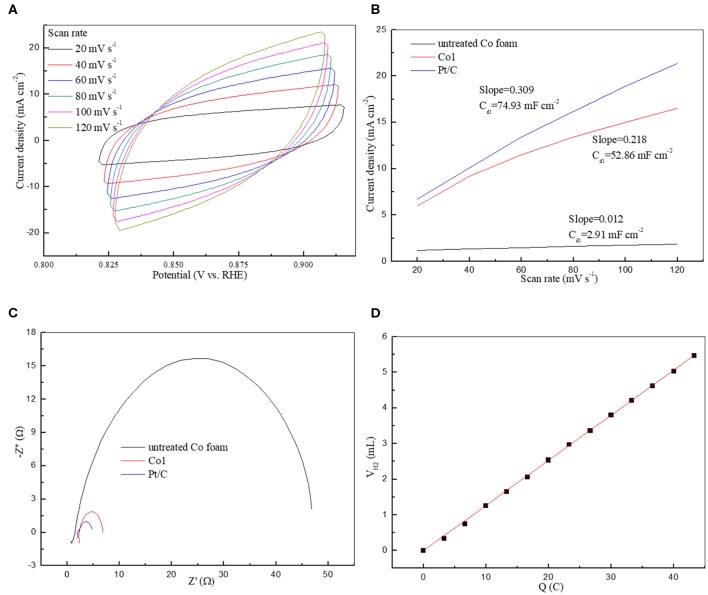
**(A)** Cyclic voltammetry of Co1 at different scan rates in the non-faradaic potential region; **(B)** Capacitive current density as a function of scan rate; **(C)** Nyquist plots of electrochemical impedance spectra in 1 M KOH; **(D)** Quantitative H_2_ measurement via water displacement.

The excellent HER performance of Co1 may be ascribed to the big surface area, giving rise to the exposure of more catalytic active sites in HER. Moreover, the electrochemical active surface area (ECSA) directly reflects electrocatalysts activity (Wei et al., 2018). The double-layer capacitance (C_dl_) at the interface between solid and liquid was also measured to reflect the effective reaction area and the quantity of active sites (Sun et al., 2016). Figure 6A and Figure S3 show the CV patterns of Co1 and other catalysts at different scan rates. As shown in Figure 6B, drawn a portrait of the current densities at the center of the testing voltage ranges along the different scan rates. In which the slopes of fitting lines are C_dl_ of the corresponding samples. The C_dl_ is in direct proportion to the electrochemical surface area of HER catalyst. The C_dl_ of Co1 is 52.86 mF cm^−2^, close to the C_dl_ of Pt/C (74.93 mF cm^−2^), and greater than the C_dl_ of the untreated cobalt foam (2.91 mF cm^−2^). The large value of C_dl_ guarantees a competitive ECSA and high HER efficiency. As shown in Table 1, using the S_BET_, the turnover frequency (TOF) of Co1 catalyst was calculated to be 0.049 s^−1^ at 100 mV. These TOF values are estimated values. This is owing to the specific active sites and non-reactive interface caused by particles contact are indeterminate. However, it is feasible to evaluate the performance of catalysts based on TOF values estimated from experimental and theoretical surface areas. EIS measurements were conducted to further determine the electrochemical reaction process of Co1 for HER. As shown in Figure 6C, Co1 affords a small charge transfer resistance (R_ct_) of 6.1 Ω, which is closed to that of Pt/C (2.9 Ω) and lower than untreated Co foam (44 Ω). Faradic efficiency would exhibit the utilization of reaction charge. As shown in Figure 6D, the quantitative H_2_ determination reveals the Faradic efficiency of Co1 catalyst is approach to 100%. It indicates that the whole charge arisen in HER procedure could be served as creating H_2_.

**Table 1:** Electrochemical parameters of different catalysts as the HER catalyst in alkaline medium.

**Electrodes**	**S_**BET**_ (m^**2**^ g^**−1**^)**	**TOF (s^**−1**^)**	**Rs (Ωcm^**2**^)**	**Rct (Ωcm^**2**^)**	**CPE (Fs^**n−1**^cm^**−2**^)**
Untreated Co foam	0.26	/	0.749	46.041	0.0012
Co1	16.15	0.049	2.355	4.472	0.0035
Pt/C	83.47	0.031	2.049	2.746	0.0047

## Conclusions

In general, the octahedral CoO_x_ catalysts are prepared on Co foam through the facile hydrothermal synthesis process. EDS and XPS results reveal that the surface of the CoO_x_ catalyst has plenty of hydroxyl groups and oxygen vacancy those further HER catalytic activity. The CoO_x_ catalyst shows excellent electrochemical performance for HER. Our strategy provides a quick and simple method to synthesize the HER catalyst used the earth-abundant element.

## Data Availability Statement

All datasets generated for this study are included in the article/Supplementary Material.

## Author Contributions

YW, RS, and JC contributed conception and design of the study and wrote sections of the manuscript. YW organized the database. RS performed the statistical analysis. JC wrote the first draft of the manuscript. All authors contributed to manuscript revision, read, and approved the submitted version.

## Conflict of Interest

RS was employed by the company Safety and Environmental Protection Division of Jilin Petrochemical Company, PetroChina, Jilin, China. YW and JC was employed by Guangdong Polytechnic Normal University, Guangdong, China.
