# Supplementary material for: Facile Synthesis of Cobalt Oxide as an Efficient Electrocatalyst for Hydrogen Evolution Reaction
Source: Front Chem. 2020 May 7;8:386. doi: 10.3389/fchem.2020.00386 (PMC7221197; doi:10.3389/fchem.2020.00386)
Supplement: Supplementary file 1 [file Table_1.docx]

**Supporting information for**

**Facile synthesis of cobalt oxide as a highly efficient electrocatalyst for hydrogen evolution reaction**

Yinbo Wu ^a*^, Ruirui Sun ^b^, Jian Cen^a, c^

^a^ Guangdong Polytechnic Normal University, Guangzhou, Guangdong 510665, China

^b^ Safety and Environmental Protection Division of Jilin Petrochemical Company, PetroChina, Jilin, Jilin 132022, China

^c^ The Key Laboratory for Smart Building Equipment Integration of Guangzhou, Guangdong 510665, China.

* Corresponding author. Tel.: +86 15989102703

E-mail addresses: [gdin_wyb@gpnu.edu.cn](mailto:gdin_wyb@gpnu.edu.cn)

1. Complementary Experimental Section
1.1 ICP test process.

Weigh 0.5 g of catalyst and place in a 250 mL glass beaker. Wet the catalyst with a small amount of water. Add 15mL HCl and stir at room temperature for about 10min. Then add 5mL HNO_3_ and heat until the sample is completely dissolved. After cooling to room temperature, transfer the solution into a 500mL volumetric flask. Dilute with water and mix well. Testing cobalt content at a wavelength of 228.615 nm.

1.2 Caculation of turn over frequencey (TOF)

Turnover frequency (TOF) is a significant performance index appraises the hydrogen evolution performance of different HER catalysts, which is the amount of H_2_ molecules generated per second per active site. TOF was obtained following the way of presented by researchers (J. Am. Chem. Soc., 134(2012)11235). Hence, the TOF about the cobalt oxide catalyst was deduced by the following equation 1:

$\frac{\# Surface sites (catalyst)}{{cm}^{2} geometric area}=\frac{\# Surface sites (flat standard)}{{cm}^{2} geometric area}\times Roughness factor$ (1)

$TOF per site=\frac{{\#Total Hydrogen Turn Overs}/{{cm}^{2} geometric area}}{{\#Surface active sites}/{{cm}^{2}geometric area}}$ (2)

**Fig. s1 XRD patterns of untreated cobalt foam**

**Fig. s2 EDS of Co1 catalyst**

**Fig. s3 Cyclic voltammetry of Co foam and Pt/C at different scan rates in 1 M KOH in the nonfaradaic potential region.**

**(a) untreated Co foam**

**(b) Pt/C**

**Fig. s4** **SEM images of Co1 after the stability measurements.**


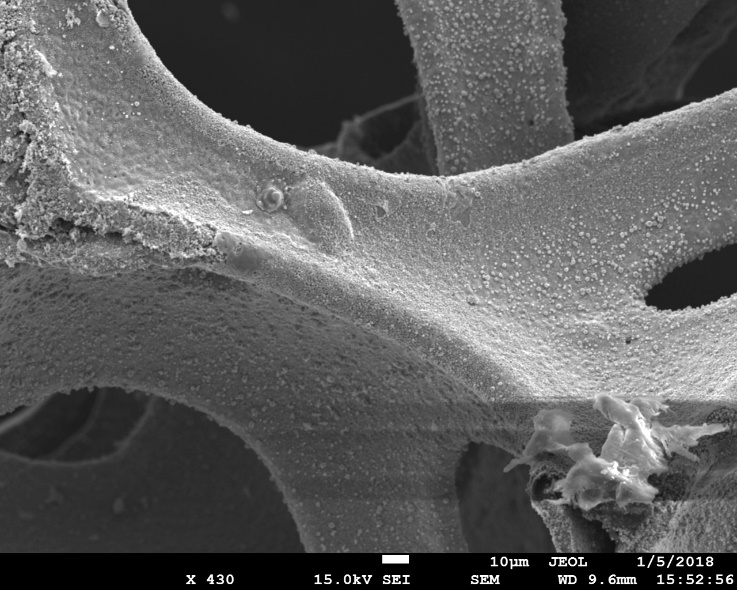

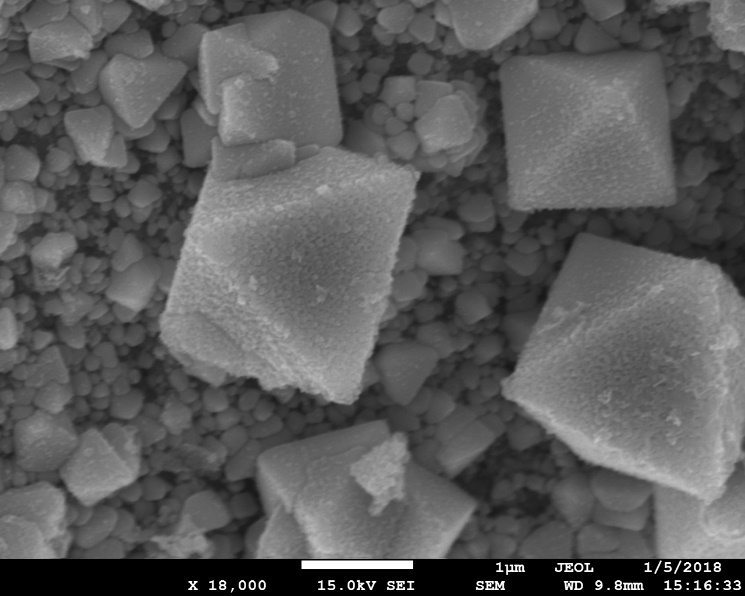


**Table S1. Performance of transition metal phosphides as HER catalyst in alkaline medium**

| Catalyst | Overpotential  at 10 mA cm^-2^  (mV) | Tafel slope  (mV dec^-1^) | Reference |
| --- | --- | --- | --- |
| Co_3_O_4_ nanosheets | 129 mV at 20 mA cm^-2^ | 49 | Nano Letters 15(2015)6015 |
| Co/CoO_x_ | 61 | 78 | ACS Applied Energy Materials 1(2018)6250 |
| CoO_x_ | 85 | 115 | Journal of The American Chemical Society 137(2015)2688 |
| CoFe-oxide | 284 mV at 50 mA·cm^−2^ | 94 | Applied Catalysis B: Environmental 258 (2019) 117968 |
| CoO_x_-N-C | 212 | 134 | Journal of Power Sources 414 (2019)333 |
| Co/CoO_x_-NPC | 259 | 99 | International Journal of Hydrogen Energy 7(2019) 3649 |
| S-CoO_x_ | 136 | 80 | Nano Energy 71 (2020) 104652 |
| Co3O4 | 140 mV at 20 mA cm^-2^ | 123 | Materials Research Bulletin 116 (2019) 98 |

**Table. S2 Element ratio value from TEM-EDS.**

| Element | Weight % | Atomic % | Uncert. % | Correction O |
| --- | --- | --- | --- | --- |
| O(K) | 34.58 | 66.01 | 0.41 | 0.47 |
| Co(K) | 65.41 | 34.03 | 0.42 | 0.93 |
